# Supplementary material for: Genome-wide CRISPR Screens in T Helper Cells Reveal Pervasive Crosstalk between Activation and Differentiation
Source: Cell. 2019 Feb 7;176(4):882–896.e18. doi: 10.1016/j.cell.2018.11.044 (PMC6370901; doi:10.1016/j.cell.2018.11.044)
Supplement: Data S2. Processed Data from All the Steps of the Analysis, Related to Figure 1 [file mmc2.zip › supplemental data/motif analysis/Pparg_homer/homerResults/motif5.similar.html]

motif5

## Information for motif5

T
A
C
G
C
A
T
G
A
T
C
G
G
C
A
T
C
T
A
G
T
C
G
A
T
G
A
C
C
T
A
G
  
Reverse Opposite:  

G
A
T
C
A
C
T
G
A
G
C
T
G
A
T
C
C
G
T
A
T
A
G
C
G
T
A
C
A
T
G
C
  

|  |  |
| --- | --- |
| p-value: | 1e-263 |
| log p-value: | -6.061e+02 |
| Information Content per bp: | 1.550 |
| Number of Target Sequences with motif | 6471.0 |
| Percentage of Target Sequences with motif | 51.69% |
| Number of Background Sequences with motif | 13060.1 |
| Percentage of Background Sequences with motif | 36.45% |
| Average Position of motif in Targets | 98.9 +/- 55.5bp |
| Average Position of motif in Background | 100.8 +/- 68.2bp |
| Strand Bias (log2 ratio + to - strand density) | -0.1 |
| Multiplicity (# of sites on avg that occur together) | 1.18 |
| Motif File: | file (matrix) reverse opposite |

### Similar de novo motifs found

|  |  |  |  |  |  |  |  |
| --- | --- | --- | --- | --- | --- | --- | --- |
| Rank | Match Score | Redundant Motif | P-value | log P-value | % of Targets | % of Background | Motif file |
| 1 | 0.825 | G C T A A C T G A G C T A G T C C T G A T A G C G T C A T A C G | 1e-205 | -473.016725 | 31.69% | 20.07% | motif file (matrix) |
| 2 | 0.655 | G T C A A C T G A C G T T A G C C T G A A G T C A C G T C G T A | 1e-91 | -210.591831 | 12.37% | 7.22% | motif file (matrix) |
| 3 | 0.662 | A C G T A T G C C G T A A G T C A G T C T C A G A G T C C G T A | 1e-68 | -157.910645 | 8.28% | 4.63% | motif file (matrix) |
| 4 | 0.642 | C G T A T C A G G A C T G T A C G T C A A G T C G T C A A T G C C A G T A C G T G A C T A T G C | 1e-38 | -88.321795 | 1.11% | 0.29% | motif file (matrix) |
